# Protective Effect of Hydrogen on Sodium Iodate-Induced Age-Related Macular Degeneration in Mice

**DOI:** 10.3389/fnagi.2018.00389

**Published:** 2018-12-04

**Authors:** Yanli Liu, Ruichan Li, Jing Xie, Jiehua Hu, Xudong Huang, Fu Ren, Lihua Li

**Affiliations:** ^1^Department of Cell Biology, Taizhou University, Taizhou, China; ^2^Information Center, Logistics College, Naval University of Engineering, Tianjin, China; ^3^Chemistry and Life College, Chengdu Normal University, Chengdu, China; ^4^Biological Anthropology Institute, Jinzhou Medical University, Jinzhou, China

**Keywords:** hydrogen, sirt1, oxidative stress, apoptosis, AMD

## Abstract

Oxidative stress is one of the main causes of AMD. Hydrogen has anti-oxidative stress and apoptotic effects on retinal injury. However, the effect of hydrogen on AMD is not clear. In this study, fundus radiography, OCT, and FFA demonstrated that HRW reduced the deposition of drusen-like structures in RPE layer, prevented retina from thinning and leakage of ocular fundus vasculature induced by NaIO_3_. ERG analysis confirmed that HRW effectively reversed the decrease of a-wave and b-wave amplitude in NaIO_3_-mice. Mechanistically, HRW greatly reduced the oxidative stress reaction through decreased MDA levels, increased SOD production, and decreased ROS content. The OGG1 expression was downregulated which is a marker of oxidative stress. Involvement of oxidative stress was confirmed using oxidative stress inhibitor ALCAR. Moreover, oxidative stress reaction was associated with expression of Sirt1 level and HRW significantly inhibited the downregulation of Sirt1 expression. This result was further confirmed with AICAR which restore Sirt1 expression and activity. In addition, NaIO_3_-induced retinal damage was related to apoptosis via caspase 8 and caspase 9, but not the caspase 3 pathways, which led to upregulation of Bax and p53, downregulation of Bcl-2, and increase in Jc-1-positive cells in mice. However, HRW effectively reversed these effects that apoptosis induced. These results suggest that HRW protects retinal functions against oxidative stress injury through inhibiting downregulation of Sirt1 and reducing retinal apoptosis. Therefore, we speculated that hydrogen administration is a promising treatment for AMD therapy.

## Introduction

AMD is the leading cause of blindness in the elderly population (Syed et al., 2012). The prevalence of AMD gradually increases with age. About 11 million people in the America suffer from visual impairment due to AMD, with approximately 170 million people in the worldwide (Pennington and DeAngelis, 2016). AMD has also become an important cause of blindness in China due to the aging population. The pathogenesis of AMD is related to many factors, such as metabolic disorders, immunity, inflammation, oxidative stress, and so on. However, oxidative stress is one of the main causes of AMD (Hernández-Zimbrón et al., 2018). During the past few decades, although aggressive and combined treatment regimens, including drugs targeting VEGF receptors, laser coagulation, gene therapy, anti-oxidants, and so on, have been used, the rate of blindness is still increasing (Holz et al., 2014; Rakoczy, 2017; Hernández-Zimbrón et al., 2018). Therefore, developing novel therapeutic agents with less toxicity and understanding their molecular mechanisms are necessary for improving AMD outcomes.

Hydrogen as a reducing agent has therapeutic effects for the reduction of oxidative stress, inflammation, and apoptosis (Xie et al., 2012; Xin et al., 2014; Shao et al., 2016; Lin et al., 2017). For example, hydrogen improves oxidant stress-induced organic injury, including acute kidney injury (Du et al., 2016), acute hepatic failure (Sun et al., 2011), chronic obstructive pulmonary disease (Liu Z. et al., 2017), and spinal cord injury (Ge et al., 2017) in rodents. Moreover, it can delay the progress of some diseases, such as diabetes and hypertension (Takeuchi et al., 2015; Guo et al., 2017). In the eye, several laboratories have shown that hydrogen exerts a neuroprotective effect on retinal ganglion cells and on retinal injury induced by light (Tian et al., 2013) in mice. For AMD studies, NaIO_3_ is used to induce oxidative stress and results in retinal function injury to mimic the characteristics of clinical AMD (Zieger and Punzo, 2016; Berkowitz et al., 2017). However, it remains unclear whether hydrogen has similar effects on AMD.

Sirt1 is a nicotinamide adenine dinucleotide-dependent protein deacetylase, which has been frequently reported to be involved in neuroprotection, cell apoptosis, cell senescence, oxidative stress, and other processes by deacetylating downstream targets (Balaiya et al., 2017). Sirt1 regulates a variety of transcription factors, including p53, FOXOs, PCG1-α, and so on (Luo et al., 2001; Brunet et al., 2004). Moreover, Sirt1 is also considered a longevity molecule to prevent against age-related diseases (Tissenbaum and Guarente, 2001; Bjørklund et al., 2018). In the eye of the rodent, immunostaining from several laboratories showed Sirt1 expression in the outer nuclear layer, inner nuclear layer, and ganglion cell layer of the retina (Jaliffa et al., 2009). Activation of Sirt1 promotes the resistance of neurons to oxidative stress and blocks damage to retinal neurons (Zeng et al., 2016). Additionally, there is more recent experimental evidence indicating that Sirt1 can directly bind to p53 to promote cell survival under stress by specifically repressing the p53-dependent apoptotic response (Brunet et al., 2004). Thus, the activation of Sirt1 may have a beneficial retinal protective effect by reducing intracellular oxidative stress and apoptosis (Zheng and Lu, 2016; Hou et al., 2017; Li et al., 2017a,b; Yao et al., 2018).

The purpose of this study was to investigate the protective role of hydrogen in AMD mice. We observed mouse fundus, retinal structure and function, as well as Sirt1 expression following hydrogen administration. We also tested the hypothesis that hydrogen can regulate retinal oxidative stress reactions and apoptosis pathways. These results suggest that hydrogen is promising treatment in AMD therapy.

## Materials and Methods

### HRW Production

Briefly, HRW was obtained by placing a metallic magnesium stick into drinking water [Mg + 2H_2_O→Mg (OH)_2_ + H_2_] and hydrogen final concentration was 0.55∼0.65 mM. The magnesium stick contained 99.9% pure metallic magnesium and natural stones in a polypropylene and ceramic container (Nakao et al., 2010).

### Animals

Healthy 8- to 10-week-old C57BL/6 male mice were purchased from the Beijing Vital River Laboratory Animal Technology Co., Ltd. (Beijing, China). The mice were maintained in a specific pathogen-free grade animal facility under a 12-h light–dark cycle. All procedures were approved by the Committee on Animal Research of Jinzhou Medical University and followed the ARRIVE guidelines pertaining to animal experimentation. NaIO_3_ (Macklin, Shanghai, China) as an inorganic substance and induced retinal damage is currently recognized as an ideal animal model for AMD research (Kannan and Hinton, 2014). The tail vein injection of NaIO_3_ to mice can cause oxidative stress reaction in the retina, which induces mice macular degeneration, which is similar to clinical AMD (Wang et al., 2014; Chowers et al., 2017).

Mice were randomly divided into three groups: control group, NaIO_3_ group, and hydrogen group. Mice in the hydrogen group were given HRW (0.1 ml/g/day, intragastric administration three times daily) by gavage for 7 days prior to a tail vein injection with NaIO_3_ at a dose of 20 mg/kg, and then HRW treatment continued for 5 days. The vehicle-treated control mice received an equal volume of 0.9% physiological saline.

ALCAR is an inhibitor of oxidative stress (Morigi et al., 2015). To further confirm that hydrogen protects against retinal damage via inhibiting NaIO_3_-induced oxidative stress, we injected intraperitoneally with ALCAR. In addition, AICAR acts as an agonist of AMPK, which activates Sirt1 after AMPK activation (Tao et al., 2017). To further confirm that hydrogen increases the expression of Sirt1 and protects against NaIO_3_-induced retinal damage, we injected intraperitoneally with AICAR in NaIO_3_ mice. So, another group of mice was randomized and pretreated with HRW for 7 days prior to an intraperitoneal injection of ALCAR (0.2 mg/g; Abcam, Cambridge, MA, USA) or AICAR (0.5 mg/g; Abcam), as described in previous studies (Morigi et al., 2015). Mice were then given a tail vein injection of NaIO_3_ (20 mg/kg) followed by continued HRW administration for 5 days.

### Fundus Photography and OCT Examination

After anesthesia with pentobarbital sodium (65 mg/kg) (Yang et al., 2012), fundus photography images were obtained using a retinal imaging system (MicrolV, Phoenix, AZ, United States). OCT images centered on the optic papilla were acquired from anesthetized animals using an OCT system (ISOCT, Optoprobe, Canada).

### FFA Assay

After anesthesia with pentobarbital sodium (65 mg/kg), sodium fluorescein (6 mg/kg) solution was injected intraperitoneally (Chung et al., 2017), and tropicamide was used. FFA examination was completed using an imaging system (OPTP-RIS, Optoprobe, Canada).

### ERG Analysis

After 12 h of dark adaptation, mice were anesthetized with pentobarbital sodium (65 mg/kg), corneal surface anesthesia was completed with oxybuprocaine hydrochloride eye drops, and tropicamide was used to dilate the pupil. Mice were placed on the operating table. A circular corneal electrode was placed on the surface of the bilateral cornea of the mouse, and a needle-shaped stainless-steel reference electrode was inserted subcutaneously behind the ear of the mouse. A needle-shaped ground electrode punctured the subcutaneous end of the mouse tail (Zhou et al., 2014). The above operation was performed under dark red light. After the baseline stabilized on the display screen, the amplitude changes of the a-wave and b-wave of 3.0 cd.s.m^-^2 ERG were recorded (ICR, Chongqing, China).

### Western Blot Analysis

Tissue homogenates were prepared from retinas of each group after adding the tissue cleavage solution and centrifugation for 30 min at 4°C and 12,000 ×*g*. The protein concentration was measured quantitatively with a BCA protein assay kit (P0010s; Beyotime; Shanghai, China). Equal amounts of protein (2 mg/ml) were separated on a 10% SDS-PAGE with an electrophoresis system. Then, the proteins were transferred to a PVDF membrane after electrophoresis and blocked with 1% fetal bovine serum and incubated in strips at 4°C with the following primary antibodies: anti-Sirt1 (ab12193; 1:1000; Abcam), anti-OGG1 (15125-1-AP; 1:1000; Proteintech; Chicago, IL, United States), anti-caspase 3 (ab44976; 1:1000; Abcam), anti-caspase 8 (ab25901; 1:1000; Abcam), anti-caspase 9 (9509S; 1:1000; CST; Massachusetts, United States), anti-P53 (ab131442; 1:1000; Abcam), anti-Bax (ab32503; 1:1000; Abcam), anti-Bcl-2 (ab32124; 1:1000; Abcam), PTG mouse anti-actin (66009-1-Ig; 1:2000; Proteintech; Chicago, IL, United States), and then with anti-rabbit and anti-mouse secondary antibodies (SA00001-2; SA00001-1; 1: 2000; Proteintech) for 2 h at room temperature. ECL emission (1705060; Bio-rad; Hercules, CA, United States) was used to visualize bands, and images were recorded through a gel imaging system. Images were scanned with ImageJ 4.0 software.

### Detection of ROS Production in the Retina

ROS levels were measured using a DHE kit (s0063, Beyotime), according to previous studies (Song et al., 2016). Briefly, mouse eyeballs were fixed and then OCT-embedded. Frozen sections (5 μm) were incubated with DHE dye in a 37°C incubator for 30 min. DHE was incubated with a superoxide anion that results in conversion to the red fluorescent compound ethidium. Fluorescence microscopy under the same exposure conditions was used to observe retinal layer ROS content. In retinal sections, the percentage of the ROS area stained with red fluorescence was normalized to the total area examined and quantified with ImageJ 4.0 analysis software.

### Jc-1 Detection of Mitochondrial Membrane Potential

The Jc-1 kit (c2006, Beyotime) was used for the detection of the mitochondrial membrane potential, according to a previous study (Mitter et al., 2014). Briefly, frozen tissue sections were incubated with a mixture of Jc-1 stain at 37°C for 20 min in an incubator. The membrane potential of the retina was measured using the same exposure conditions with a fluorescence microscope. The percentage of the apoptosis area stained with green fluorescence was normalized to the total area examined and quantified with ImageJ 4.0 software.

### Oxidative Stress Levels Detected

Retina tissues were weighed and washed in PBS and then homogenized immediately in 10 volumes of PBS at 37°C. After centrifugation, supernatants were collected and stored at -80°C. The levels of activated SOD and oxidative stress product MDA were measured according to the instructions of the kit (A001-3, A003-1; Nanjing Jiancheng Bioengineering Institute, Nanjing, China). The absorbance was read on a microplate reader (Denley Dragon, Wellscan MK3, Thermo, Finland), and the concentrations were calculated based on a standardized curve.

### Statistical Analysis

All data are expressed as the mean ± SEM, and one-way ANOVA was used to compare differences between the groups. The LSD test was used to compare multiple pairs of means. Comparison between two groups was done with a *t*-test. *P* < 0.05 was considered statistically significant.

## Results

### HRW Has Protective Effects on NaIO_3_-Induced Retinal Damage

**FIGURE 1:**
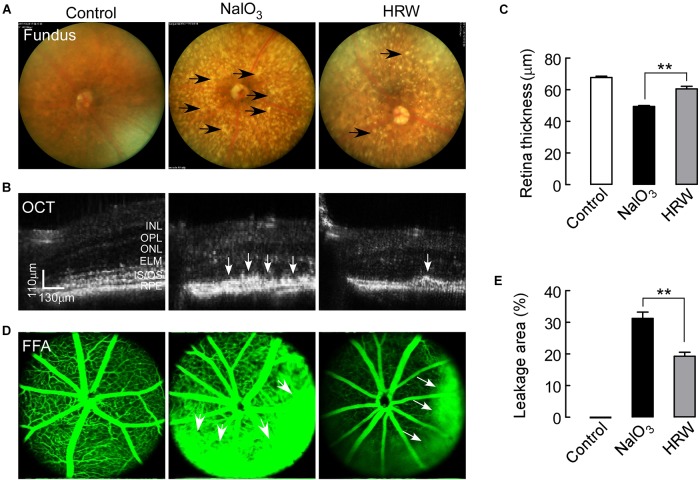
Effects of HRW on NaIO_3_-induced fundus and retinal impairment in mice. After cotreatment with HRW (0.1 ml/g/day, i.g.) and NaIO_3_ (20 mg/kg, i.v.) for 12 days, retinas were evaluated with the fundus photography, OCT examination, and FFA examination. **(A)** Representative images of fundus photography. The black arrow represents yellow-white, drusen-like structures. **(B)** Representative images of OCT in the mouse retina and **(C)** quantification of retinal thickness. White arrows indicate areas of hyperreflexia in the RPE area. **(D)** Representative images of FFA and **(E)** quantification of the leakage area. White arrowheads represent the leaking area. RPE, retinal pigment epithelium; IS/OS, inner segment/outer segment; ONL, outer nuclear layer; OPL, outer plexiform layer; INL, inner nuclear layer. Values are presented as the mean ± SEM, *n* = 5, ^∗∗^*P* < 0. 01 vs. the NaIO_3_ group.

To assess the protective effect of HRW treatment on NaIO_3_-induced retinal damage in mice, we performed eye functional examinations in NaIO_3_ mice with or without HRW treatment. Fundus photography revealed a large number of yellow-white, drusen-like structures in the fundus of NaIO_3_-treated mice. We observed a significant reduction in yellow-white deposits on the fundus after HRW treatment (Figure 1A). By OCT, we found that the RPE layer of mice in the NaIO_3_ group developed a large number of high reflex zones and the retina became thin (Figure 1B). However, HRW prevented retinal thinning and reduced the number of high reflex zones in the RPE layer in mice that received HRW-treatment (Figure 1C, *P* < 0.01). To further confirm the damage of NaIO_3_ to the retina, we examined the retinal vascular integrity. The results of the FFA assay showed that retinal blood vessel leakage appeared in NaIO_3_-treated mice (Figure 1D), but the retinal leakage area in HRW-treated mice was much smaller than that in NaIO_3_ mice (Figure 1E, *P* < 0.01). These results confirm the protective effect of HRW on retinal injury.

**FIGURE 2:**
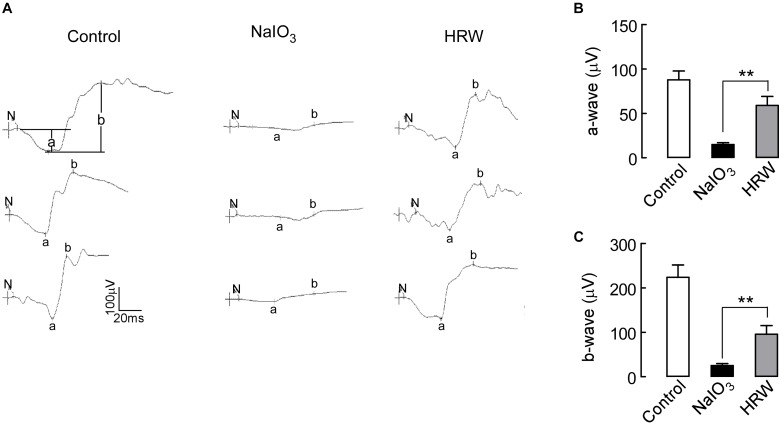
Effects of HRW on NaIO_3_-induced visual function impairment in mice. **(A)** ERG map of dark adaptation 3.0 in control, NaIO_3_, and HRW groups. N represents the start of stimulation, a represents the amplitude of the a-wave, and b represents the b-wave amplitude. **(B,C)** Quantification of the a-wave and b-wave amplitudes, respectively. Values are presented as the mean ± SEM, *n* = 10, ^∗∗^*P* < 0.01 vs. the NaIO_3_ group.

Studies have shown that NaIO_3_ can damage RPE cells and retinal visual function (Zieger and Punzo, 2016). We assessed the effect of HRW on retinal function using an ERG assay in mice. The a-wave reflects the function of the cone cells and the rod cells, and the b-wave represents the function of the bipolar cells. In the dark-adapted 3.0 ERG, a- and b-wave amplitudes were significantly increased in the HRW-treated mice compared to those in the NaIO_3_ mice, reflecting the recovery of visual function in HRW-treated mice and the resistance to NaIO_3_-induced functional visual damage by HRW (Figure 2; *P* < 0.05). This result suggested that HRW can protect the retina from NaIO_3_-induced damage in mice.

### HRW Reduces the Oxidative Stress Reaction in the Mouse Retina After NaIO_3_ Treatment

**FIGURE 3:**
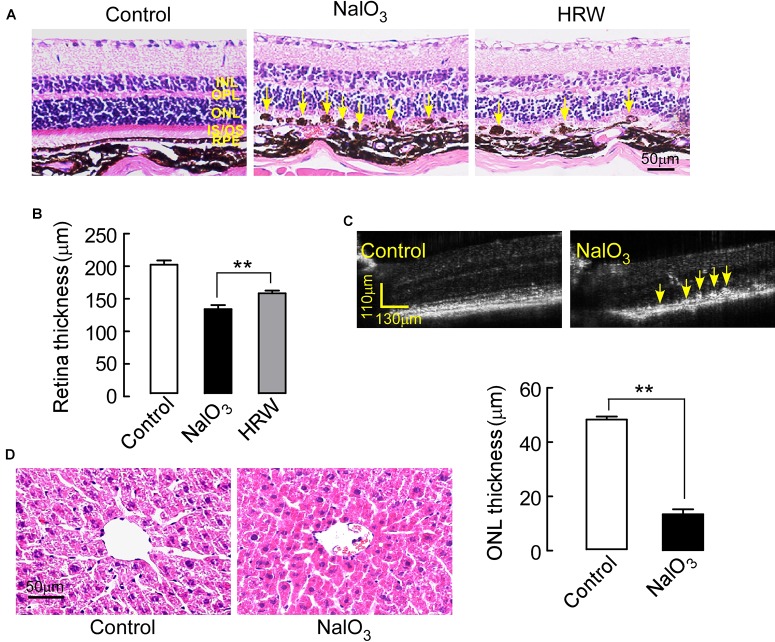
Effects of HRW on NaIO_3_-induced retinal morphological impairment in mice. Mouse eyeballs were harvested after cotreatment with HRW and NaIO_3_ for 12 days. **(A)** Representative images of H&E-stained eyeball sections from control and NaIO_3_ mice treated with or without HRW. Yellow arrows indicate drusen-like melanin depositions in the RPE layer. **(B)** Quantitative analysis of retinal thickness via H&E-stained eyeball sections. The values are expressed as the mean ± SEM, *n* = 10. **(C)** Representative images of OCT in the mouse retina in control and NaIO_3_ groups. Yellow arrows point toward areas of hyperreflexia in the RPE. **(D)** H&E staining of the liver after 5 days of NaIO_3_ injection. *n* = 5, ^∗∗^*P* < 0.01 vs. the NaIO_3_ group.

The mechanism of NaIO_3_-induced retinal damage is associated with oxidative stress, but whether the protective effect of HRW on the retina is through a reduction in oxidative stress was examined. First, we detected NaIO_3_-induced retinal damage with H&E staining. It clearly showed that a large amount of melanin deposition appears in the RPE layer retinas from NaIO_3_ mice compared to retinas from control mice. The amount of the black sediment on the retina in HRW-treated mice is greatly reduced compared to the amount in NaIO_3_ mice (Figure 3A). Moreover, the retinal layers in NaIO_3_ mice were thinner than those of the control mice. However, HRW prevents retinal thinning (Figure 3B, *P* < 0.01). In addition, the thickness of the outer nuclear layer in NaIO_3_ mice is significantly reduced compared to that in control mice (Figure 3C, *P* < 0.01), which indicated that NaIO_3_ caused retinal injury. In addition, we assessed the toxic effect of NaIO_3_ on the liver using H&E staining. No changes were observed in liver sections between the three groups (Figure 3D), suggesting that NaIO_3_ selectively damages the eyeball.

**FIGURE 4:**
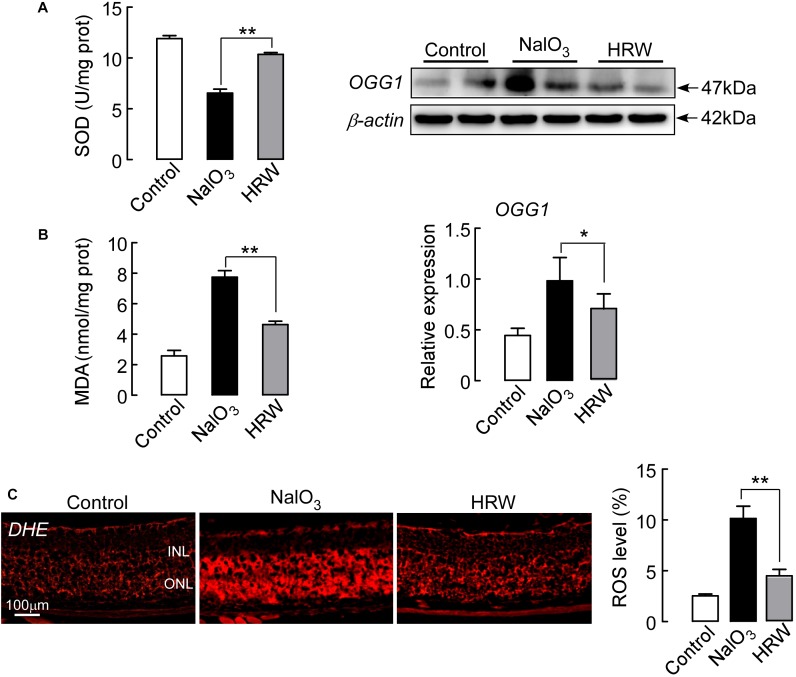
Effect of HRW on NaIO_3_-exerted retinal oxidative stress in mice. Retinal tissue collection after cotreatment with HRW and NaIO_3_ for 12 days. **(A)** The level of SOD and MDA in the retina was analyzed by SOD and MDA kits. **(B)** Representative blots and densitometry data of OGG1 expression in the retinas from NaIO_3_ mice with and without HRW treatment. **(C)** The activity of ROS was measured by DHE staining, the red staining is positive for ROS content. Data represent the mean ± SEM of three independent experiments, *n* = 3, ^∗^*P* < 0.05, ^∗∗^*P* < 0.01 vs. the NaIO_3_ group.

Next, we observed the oxidative stress reaction in the retina. As Figure 4A shows, HRW significantly increased the activity of the oxidative stress inhibitory enzyme SOD, whereas it decreased MDA content in NaIO_3_ mice. To further confirm this result, we examined the expression level of OGG1, an oxidative stress marker protein, in the retina. The expression of OGG1 in NaIO_3_ mice is greatly increased compared to control expression. However, HRW significantly reduced the NaIO_3_-induced increase in OGG1 expression (Figure 4B; *P* < 0.05). DHE staining, which reflects the ROS content in the retina, showed a marked decrease in red fluorescence of ROS-stained retinal tissue in HRW-treated mice compared to the retinas of NaIO_3_ mice (Figure 4C; *P* < 0.01). These results suggest that HRW can attenuate the oxidative stress reaction by NaIO_3_-induction in the mouse retina.

### HRW Inhibits Downregulation of Oxidative Stress-Induced Sirt1 in Mice

**FIGURE 5:**
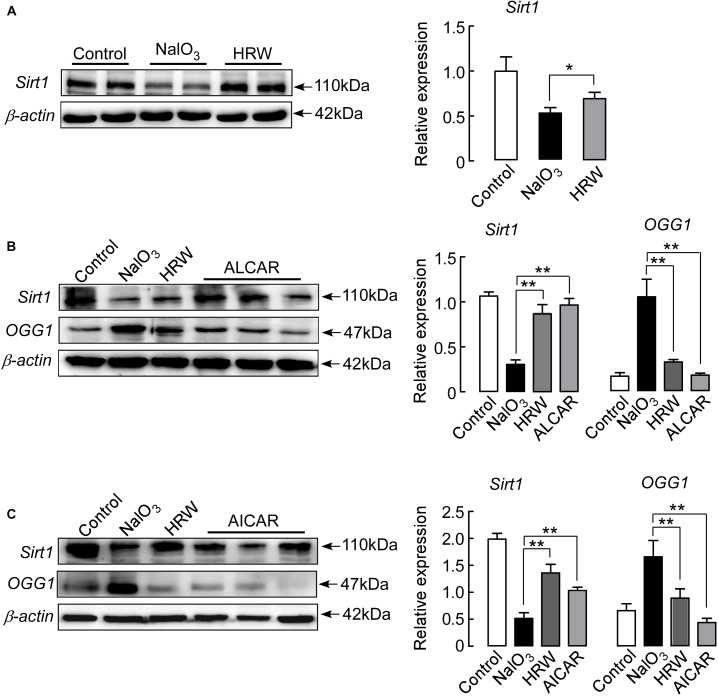
HRW upregulates Sirt1 expression in the retina by inhibiting oxidative stress. **(A)** Representative blots and quantitative analysis of Sirt1 protein expression at 5 days after NaIO_3_ injection. Mice were pretreated with ALCAR (0.2 mg/g, i.p.) **(B)** or AICAR (0.5 mg/g, i.p.) **(C)** before NaIO_3_ injection. The expression of Sirt1 and OGG1 were measured by Western blotting. Data represent the mean ± SEM of three independent experiments, *n* = 3–6, ^∗^*P* < 0.05, ^∗∗^*P* < 0.01 vs. the NaIO_3_ group.

Sirt1 plays an important role in various retinal diseases, including anti-oxidant and anti-apoptotic effects in mice (Han et al., 2017). In this study, we assessed whether HRW reduced the oxidative stress reaction through the regulation of Sirt1 expression. Western blotting indicated that Sirt1 expression was significantly downregulated in NaIO_3_ mice, and HRW treatment reversed the NaIO_3_-induced downregulation of Sirt1 expression (Figure 5A; *P* < 0.05).

We next tested whether HRW inhibits the downregulation of Sirt1 through a reduction in oxidative stress. Mice were injected with ALCAR, an inhibitor of oxidative stress, before NaIO_3_ treatment, and then we measured Sirt1 and OGG1 expression. We found that ALCAR significantly reduced the NaIO_3_-induced downregulation of Sirt1 and increased OGG1 expression in the mouse retina (Figure 5B; *P* < 0.01). These results confirm that HRW indeed inhibits the downregulation of Sirt1 expression by decreasing oxidative stress.

In addition, we further analyzed whether HRW protects NaIO_3_-induced retinal damage through the regulation of Sirt1 expression. Mice were treated with the Sirt1 indirect activator AICAR prior to NaIO_3_ administration. We found that AICAR treatment significantly increased the expression of Sirt1 in NaIO_3_ mice (Figure 5C; *P* < 0.01). At the same time, we measured the expression of OGG1. AICAR could also inhibit the NaIO_3_-induced OGG1 upregulation (Figure 5C; *P* < 0.01), suggesting that HRW could inhibit the downregulation of oxidative stress-induced Sirt1 expression.

### HRW Inhibits NaIO_3_-Induced Apoptosis Through the Regulation of Sirt1 Expression in the Mouse Retina

**FIGURE 6:**
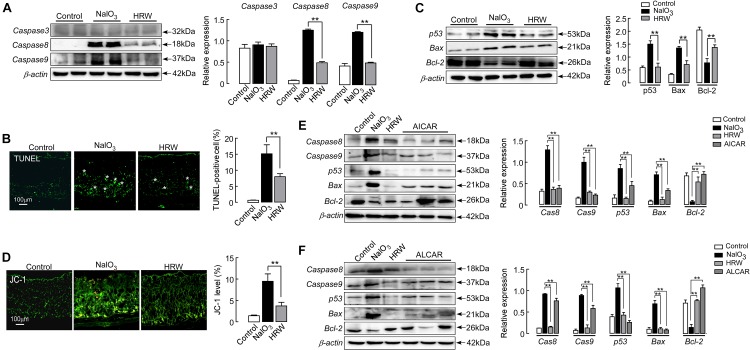
HRW reduces apoptosis in the retina by upregulating Sirt1. **(A)** Representative blot and quantitative analysis of caspase 3, caspase 8, and caspase 9 protein expression levels at 5 days after NaIO_3_ injection with or without HRW administration. **(B)** Representative images of TUNEL-stained retinal sections from control and NaIO_3_ mice with or without HRW administration. TUNEL-positive cells are shown by asterisks. Data represent the mean ± SEM of three independent experiments. *n* = 10. **(C)** Representative blot and the quantitative analysis of protein expression for p53, Bax, and Bcl-2 expression in the retina. *n* = 4. **(D)** Representative images of Jc-1-stained retinal sections and the quantification of green fluorescence intensity in retinal sections. *n* = 5. Representative Western blot and the quantitative analysis of caspase 8, caspase 9, p53, Bax, and Bcl-2 protein expression after ALCAR **(E)** or AICAR **(F)** injection in retinas from NaIO_3_ mice. Values were presented as the mean ± SEM. *n* = 3–6, ^∗∗^*P* < 0.01 vs. the NaIO_3_ group.

Studies have shown that NaIO_3_ induces retinal damage by activating the pathway of apoptosis (Jaliffa et al., 2009; Balmer et al., 2015; Zeng et al., 2016). We first examined apoptosis in the retina of NaIO_3_ mice. TUNEL staining showed that TUNEL-positive cells (green) were mainly concentrated in the outer nuclear layer of the retina in the NaIO_3_ mice. HRW greatly decreased the number of TUNEL-positive cells in the retina (Figure 6B; *P* < 0.01). Further, we analyzed the expression of apoptosis pathway-related proteins in the mouse retina. However, no change was observed in the expression of caspase 3 between mice in all groups (*P* > 0.05). HRW significantly reduced the expression of caspase 8 (*P* < 0.01), caspase 9 (Figure 6A; *P* < 0.01), p53, and Bax, whereas it increased Bcl-2 expression (Figure 6C; *P* < 0.01) in NaIO_3_ mice. Additionally, Jc-1 can be used to detect early apoptosis by indicating changes in the mitochondrial membrane potential (Wang et al., 2015). As shown in Figure 6D, early apoptotic cells (green) are abundantly apparent in the retina of NaIO_3_ mice, and the green fluorescence intensity of Jc-1-staining in HRW-treated mice is significantly decreased (*P* < 0.01). These results suggest that HRW could inhibit NaIO_3_-induced retinal apoptosis via caspase 8 and caspase 9 apoptotic pathways.

Sirt1 has anti-apoptotic and anti-oxidative stress effects in the rat (Yang et al., 2013; Qi et al., 2015). To demonstrate the anti-apoptotic effect of Sirt1, we examined the expression of apoptosis-related regulators after administration of the Sirt1 indirect agonist AICAR in mice. AICAR significantly inhibited the expression of caspase 8, caspase 9, p53, and Bax, but increased the expression of Bcl-2 in NaIO_3_ mice (Figure 6E; *P* < 0.01). These results confirm that HRW has an anti-apoptotic effect through the increase of Sirt1 expression in NaIO_3_-induced retinal damage.

In addition, we further determined the relationship between oxidative stress and apoptosis using an inhibitor of oxidative stress in NaIO_3_ mice. After mice were given ALCAR, we examined the expression of proapoptotic proteins (caspase 8, caspase 9, p53, and Bax) and the anti-apoptotic protein Bcl-2. ALCAR significantly inhibited the upregulation of proapoptotic protein expression and downregulation of Bcl-2 protein in NaIO_3_ mice (Figure 6F; *P* < 0.01). These results suggest that HRW could effectively inhibit the downregulation of Sirt1 expression through antioxidative stress and has a further antiapoptotic effect on NaIO_3_-induced retinal injury.

## Discussion

Previous studies have indicated that hydrogen has ideal therapeutic effects for oxidant stress and inflammation (Xin et al., 2014; Lin et al., 2017). Although some studies have demonstrated the anti-apoptosis and anti-oxidative stress effects of hydrogen on light-induced retinal injury in the rodent (Takeuchi et al., 2015), the effect of hydrogen on AMD is still unknown. In the present study, we investigated the retinal protective effects and mechanisms of action of hydrogen in NaIO_3_-induced mice. Our data demonstrated that HRW could reduce retinal damage through a decreased oxidative stress reaction and inhibit the NaIO_3_-induced downregulation of Sirt1 expression (Figures 4, 5A). Involvement of oxidative stress and the Sirt1 protein was confirmed using the oxidative stress inhibitor ALCAR and the Sirt1 activator AICAR (Figures 5B,C). Moreover, HRW inhibits apoptosis via the caspase 8 and 9 pathways, but not the caspase 3 pathway, in NaIO_3_ mice retinas (Figure 6). Interestingly, we found that the injury of NaIO_3_ is tissue selective and NaIO_3_ mouse liver sections did not display a significant toxic effect with H&E staining (Figure 3).

HRW can improve the quality of human life and prevent the occurrence of diseases (Mizuno et al., 2018). HRW also can protect the retina from injury in eye diseases in rats (Qi et al., 2015; Chen et al., 2016). We investigated the protective effect of HRW in AMD mice. After mice were given HRW and NaIO_3_, through fundus photography and OCT we found that HRW reduced the deposition of yellow-white, drusen-like structures induced by NaIO_3_ and prevented the thinning of the retina (Figures 1A,B). Consistently, it reduced the degree of retinal degeneration. We further found that HRW reduced the area of fundus leakage induced by NaIO_3_ and maintained the integrity of fundus vessels (Figure 1D). Further, when we observed the retinal function by ERG, we found that HRW increased the survival of rod and cone cells and reversed the degree of visual function impairment by NaIO_3_ (Figure 2).

Oxidative stress is one of the many factors in the pathogenesis of AMD (Hernández-Zimbrón et al., 2018). NaIO_3_ induces retinal injury through oxidative stress (Kannan and Hinton, 2014; Berkowitz et al., 2017). As hydroxyl radicals, hydrogen can combine with excess oxygen free radicals, causing inhibition of the oxidative stress reaction by decreasing MDA content and upregulating SOD (Wu et al., 2017). There is growing evidence that hydrogen plays important roles in ROS reduction (Liu et al., 2014). After administration of NaIO_3_, the RPE layer of the retina became thinner or even ruptured. There was a large amount of black deposits, similar to drusen, between the RPE layer and Bruch’s membrane (Figure 3A). The ONL membrane and the overall thickness of the retina were decreased (Figure 3C). HRW can reduce retinal morphological damage induced by NaIO_3_ (Figure 3B). The index of oxidative stress showed that HRW decreased the content of MDA in oxidative stress products and prevented the decrease of SOD activity with the oxidative stress inhibitor after NaIO_3_ treatment (Figure 4A). In addition, we observed the amount of ROS induced by NaIO_3_ was reduced by HRW because there was a decreased number of ROS-positive cell as shown by the fluorescent intensity of DHE staining in the retina (Figure 4C). Further, to determine that HRW can inhibit oxidative stress, we measured the expression of the oxidative stress marker protein OGG1. The results of our study showed that hydrogen significantly decreased oxidant levels induced by NaIO_3_ (Figure 4B).

As a deacetylase, Sirt1 is closely related to apoptosis and oxidative stress (Chen et al., 2016). Sirt1 plays a protective role in regulating apoptosis in the retina. With increasing age, the expression of Sirt1 increases in the retina, thus increasing the resistance of the retina to the outside damage (Zeng and Yang, 2015). Hydrogen can activate Sirt1 and protect against damage from diseases, and hydrogen can inhibit oxidative stress and regulate the expression of Sirt1. In our studies, the expression of Sirt1 was downregulated by NaIO_3_ administration (Figure 5A). However, HRW could elevate the expression of Sirt1 by inhibiting oxidative stress (Figure 5B). Furthermore, oxidative stress inhibitors and Sirt1 activators caused an increase in Sirt1 levels and decrease in OGG1 levels, suggesting that the antioxidant stress of HRW can prevent the downregulation of Sirt1 induced by NaIO_3_ (Figure 5C). Our study indicated that hydrogen directly mediates the expression of Sirt1 by anti-oxidative stress.

Studies have shown that hydrogen inhibits apoptosis by activating Bcl-2 and inhibiting Bax and caspase 3 (He et al., 2016; Chen et al., 2017). Sirt1 inhibits apoptosis by upregulating the expression of Bax (Liu S. et al., 2017). Sirt1 can inhibit apoptosis by regulating the Bcl-2 family and caspase 3 (Gu et al., 2018). Some studies suggest that oxidative stress inhibits the activity of Sirt1 and then regulates p53-dependent apoptosis (He et al., 2017). In this study, we used TUNEL staining to demonstrate that HRW has an anti-apoptotic effect (Figure 6B). HRW also reduced the expression of caspase 8 and caspase 9 but did not affect caspase 3 expression (Figure 6A). Moreover, our studies further found that HRW decreased the expression of p53 and Bax, but increased the expression of Bcl-2 after NaIO_3_ administration (Figure 6C), suggesting that the anti-apoptotic effect of hydrogen was through the caspase 8 and caspase 9 pathways. The measurement of the mitochondrial membrane potential also confirmed the role of the anti-apoptotic effect of HRW (Figure 6D). The HRW inhibition of apoptosis was mimicked by oxidative stress inhibitors and Sirt1 activators (Figures 6E,F). These results suggest that hydrogen could promote cell survival in the retina after oxidative stress, and hydrogen inhibits apoptosis by regulating the expression of Sirt1. Our results suggest that hydrogen inhibits oxidative stress and the downregulation of Sirt1 induced by NaIO_3_ and plays a role in preventing apoptosis.

Molecular hydrogen as a medical gas could be used in antioxidant therapy in numerous human diseases (Kurokawa et al., 2015). The primary advantage of HRW is that safe means of delivering hydrogen (Chen et al., 2016). Hydrogen is a colorless, transparent, odorless, and tasteless gas. It is insoluble in water as the lightest gas. *In vitro* experiments were carried out under normal pressure. Because of the characteristics of hydrogen and the limitations of our current laboratory conditions, in our studies, AMD animal model was used to confirm the therapeutic effect of hydrogen on AMD. At the same time, we used oxidative stress inhibitors and Sirt1 activators to confirm the role of hydrogen in anti-oxidative stress and anti-apoptosis of AMD. We will conduct *in vitro* experiments if conditions permit to further support our point of view.

In summary, we found that hydrogen can reverse the production and progress of drusen, improve the function of the optic nerve and the integrity of fundus vessels. Hydrogen regulates the expression of Sirt1 in the retina by inhibiting oxidative stress. Hydrogen inhibits the downregulation of Sirt1 induced by NaIO_3_ and inhibits apoptosis induced by NaIO_3_ via regulation of Sirt1 expression. Our findings suggest that hydrogen is an effective therapeutic strategy for the pathogenesis of oxidative stress in AMD.

## Author Contributions

LL and YL conceived and designed the experiments. YL, RL, JH, JX, XH, and FR performed the experiments. LL, YL, RL, and FR analyzed the data. LL contributed reagents, materials, and analysis tools. LL and YL wrote the paper. XH contributed the process of revised article.

## Conflict of Interest Statement

The authors declare that the research was conducted in the absence of any commercial or financial relationships that could be construed as a potential conflict of interest.

## Abbreviations

AICAR: 5-aminoimidazole-4-carboxamide1-β-D-ribofuranoside
ALCAR: acetyl-L-carnitine
AMD: age-related macular degeneration
AMPK: AMP-activated protein kinase
ANOVA: analysis of variance
Bax: BCL2-associated X protein
Bcl-2: B-cell lymphoma-2
DHE: dihydroethidium
ECL: enhanced chemiluminescence
ERG: electroretinography
FFA: fundus fluorescein angiography
FOXOs: Forkhead box protein Os
H&E: hematoxylin and eosin
HRW: hydrogen-rich water
Jc-1: mitochondrial membrane potential assay kit with JC-1
LSD: least significant difference
MDA: malondialdehyde
NaIO_3_: sodium iodate
OCT: optical correlation tomography
OGG1: 8-oxoguanine DNA glycosylase
PBS: phosphate buffer saline
PCG1-α: receptor γ coactivator-1 α
PVDF: polyvinylidene difluoride membrane
ROS: reactive oxygen species
RPE: retinal pigment epithelium
SDS-PAGE: sodium dodecyl sulfate polyacrylamide gel electrophoresis
SEM: standard error of the mean
Sirt1: silent information regulator 2 homolog 1
SOD: superoxide dismutase
TUNEL: TdT-mediated dUTP Nick-end labeling
VEGF: vascular endothelial growth factor
